# Porous Carbon Networks Derived From Graphitic Carbon Nitride for Efficient Oxygen Reduction Reaction

**DOI:** 10.1186/s11671-019-3073-0

**Published:** 2019-07-24

**Authors:** Chenxia Li, Xuesong Li, Xiaojuan Sun, Xueyu Zhang, Lianfeng Duan, Xijia Yang, Liying Wang, Wei Lü

**Affiliations:** 1 Key Laboratory of Advanced Structural Materials, Ministry of Education & Advanced Institute of Materials Science, Changchun University of Technology, Changchun, Changchun, 130012 China; 2 State Key Laboratory of Luminescence and Applications, Changchun Institute of Optics, Fine Mechanics and Physics, Chinese Academy of Sciences, Changchun, 130012 China

**Keywords:** Mesoporous carbon, Carbon-based materials, Metal free, Oxygen reduction reaction

## Abstract

Great efforts have been dedicated to finding economic and efficient oxygen reduction reaction (ORR) for fuel cell technology. Among various catalysts, N-doped carbon-based nanomaterials have attracted much attention due to low-cost, noble metal free, and good durability. Herein, we developed a facile and economic strategy to prepare nitrogen-doped carbon networks for efficient ORR application. The g-C_3_N_4_ is used as the template and N source, and dopamine is used as the carbon source. By simple hydrothermal treatment and sintering, N-doped carbon network structures with high specific surface area, effective ORR activity, and superior durability could be acquired. The present strategy is free of involving generally multistep, poisonous reagents, and complication of removing template for fabrication of 3D carbon structures.

**Electronic supplementary material:**

The online version of this article (10.1186/s11671-019-3073-0) contains supplementary material, which is available to authorized users.

## Background

The oxygen reduction reaction (ORR) is a crucial step for further development of clean energy conversion strategies such as fuel cells and metal-air batteries [1–3]. The traditional Pt-based cathode materials for ORR are generally suffered from high cost, limited stability, and poor tolerance to methanol [1, 4–8]. Therefore, to develop a low cost, highly active, durable material towards ORR has received great attention [9, 10]. Numerous efforts have been dedicated to searching replacement for Pt-based electrocatalysts, such as transition-metal catalysts [5, 11–13], and carbon-based nanomaterials [4, 8, 13–16].

Notably, as metal-free electrocatalysts, carbon-based nanomaterials are promising materials for ORR due to good durability, noble metal free, and low-cost [17]. By elegant design of material system, 3D porous carbon structures could be achieved and provided high specific area and pore volume, which is extremely important for efficient ORR [7, 18]. Desirable three-dimensional carbon structures generally derived from various templates including ice, silica, and polystyrene [19]. The fabrication of 3D carbon structure generally involved multistep, poisonous reagents, and complication of removing template [6, 20, 21]. Thus, facile preparation strategy is still a main obstacle. In addition, the introduction of N atoms in carbon materials significantly enhances electrocatalytic activity thus inducing efficient ORR [22, 23]. Great efforts have reported N-doped carbon materials by introducing nitrogen-rich source such as melamine [24, 25], urea [26], dopamine [27], and pyrrole followed by sintering. For ORR applications, a facile way to realize porous structure and efficient N doping is still highly desired.

Herein, we developed a strategy to prepare nitrogen-doped carbon networks for efficient ORR application using metal-free graphitic carbon nitride (g-C_3_N_4_) and dopamine (DA) as N source and C source, respectively. Metal-free g-C_3_N_4_ has been intensively investigated due to its potential application for photocatalysis [9, 28–30] and ORR [30–33] etc. The N-doped materials could be achieved using g-C_3_N_4_ as the N source due to its high N content [20, 23, 34, 35]. The g-C_3_N_4_ is a typical two-dimensional conjugated polymer material [36, 37]. It has received extensive attention as an inexpensive, metal-free, visible light-responsive photocatalyst [38, 39]. The g-C_3_N_4_ has excellent electronic band structure, surface functionalization modification, and high physical and chemical stability and is non-toxic and rich in raw materials [40–42]. In addition, the nitrogen content is high, making it one of the known N-rich compounds [43]. The most important thing is that it has a variety of 2D or 3D structures that can be obtained by controlling the synthesis conditions [44–46]. Nitrogen-doped carbon materials generally have a synthesis temperature above 800 °C, which satisfies the requirements for removing the template [47]. Therefore, it is possible to utilize g-C_3_N_4_ that only contains carbon and nitrogen elements to synthesize N-doped carbon materials [48]. In the present work, g-C_3_N_4_ is used as a template and N source simultaneously to prepare porous carbon structures with high specific surface area (954 m^2^ g^−1^) and 5.71% N content is achieved, which exhibits comparable ORR activity, superior durability, and methanol tolerance to Pt/C reference electrocatalyst.

## Methods

### Materials

Potassium hydroxide (KOH) and potassium chloride (KCl) were obtained from Sinopharm Chemical Reagent Co., Ltd. Potassium hexacyanoferrate (K_3_[Fe(CN)_6_]) were obtained from Tianjin Yongsheng Fine Chemical Co., Ltd. Urea were obtained from Beijing Chemical Corp. All of the above drugs are analytically pure. Naifon® perfluorinated solution (5 wt. % in mixture of lower aliphatic alcohols and water, contains 45% water) was purchased from Sigma-Aldrich.

### Synthesis of g-C_3_N_4_ Template

Typically, 15 g of urea in 100 mL crucible was kept at 550 °C for 4 h. The g-C_3_N_4_ was acquired and grounded to light yellow powder for later use after cooling to room temperature.

### Synthesis of g-C3N4@dopamine Precursors

0.5 g g-C_3_N_4_ was dispersed in 20 mL DA solution. The concentration of DA was 0.3 M. The mixture was ultrasonicated for 2 h and transferred into an autoclave followed by heating at 120 °C for 10 h. The resulted sample was centrifuged and washed followed by drying at 80 °C overnight. Three heating temperatures of 120 °C, 140 °C, and160 °C were used for preparing g-C_3_N_4_/PDA precursors, and the corresponding samples were named g-C_3_N_4_/PDA-120, g-C_3_N_4_/PDA-140, and g-C_3_N_4_/P DA-160, respectively.

### Preparation of Nitrogen-Doped 2D Carbon Materials

**Scheme 1:**
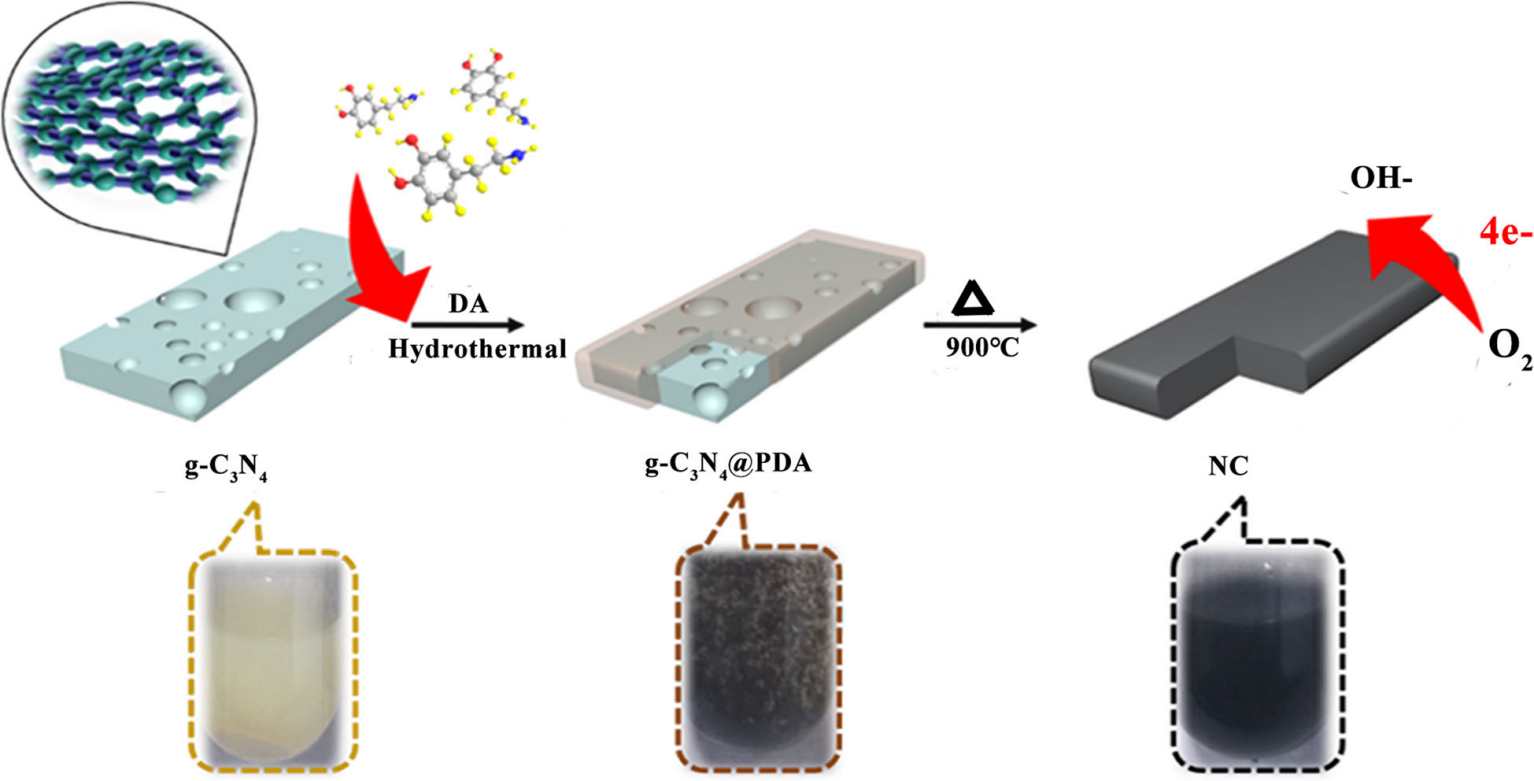
The synthetic process of NC-T electrocatalysts

The precursors of g-C_3_N_4_/PDA-120, g-C_3_N_4_/PDA-140, and g-C_3_N_4_/PDA-160 were heated to 900 °C for 2 h in nitrogen atmosphere. After cooling to room temperature, nitrogen-doped porous carbon samples named NC-120, NC-140, and NC-160 (NC-T) were synthesized. However, the attempt for further decreasing heating temperature to 100 °C induced very poor coating of DA on g-C_3_N_4_, which resulted in low yield after sintering at 900 °C. Therefore, three temperatures of 120 °C, 140 °C, and 160 °C were chosen for further investigation. The synthesis process of nitrogen-doped porous carbon samples is shown in Scheme 1.

### Electrochemical Measurement

Electrochemical analysis was fulfilled by the DyneChem electrochemical workstation, and Ag/AgCl and platinum are used as reference electrode and counter electrode, respectively. The cyclic voltampis was tested in 0.1 M potassium hydroxide solution. The glass carbon electrode (GCE) was polished and washed before using. To prepare the working electrodes, aliquots of 5 μL and 2.5 mg/mL NC-120, NC-140, NC-160, Pt/C solutions were dipped on to GCE for further test.

### Characterization

The structure and chemical composition of the NC-T was analyzed by X-ray diffraction (XRD) (D-MAX II A X-ray diffractometer), transmission electron microscopy (TEM) (Tecnai F20), scanning electron microscope (SEM) (JEOL7610), fourier transform infrared (FT-IR) (Nicolet iS50) spectra, X-ray photoelectron spectroscopy (XPS) (Kratos Axis UltraDLD), and Raman (Horiba, Japan); N2 adsorption-desorption (77 K) isotherms were carried out on a Micromeritics ASAP 2020 instrument (MICROSENSOR, USA).

## Results and Discussion

### SEM and TEM Characterization

**Fig. 1:**
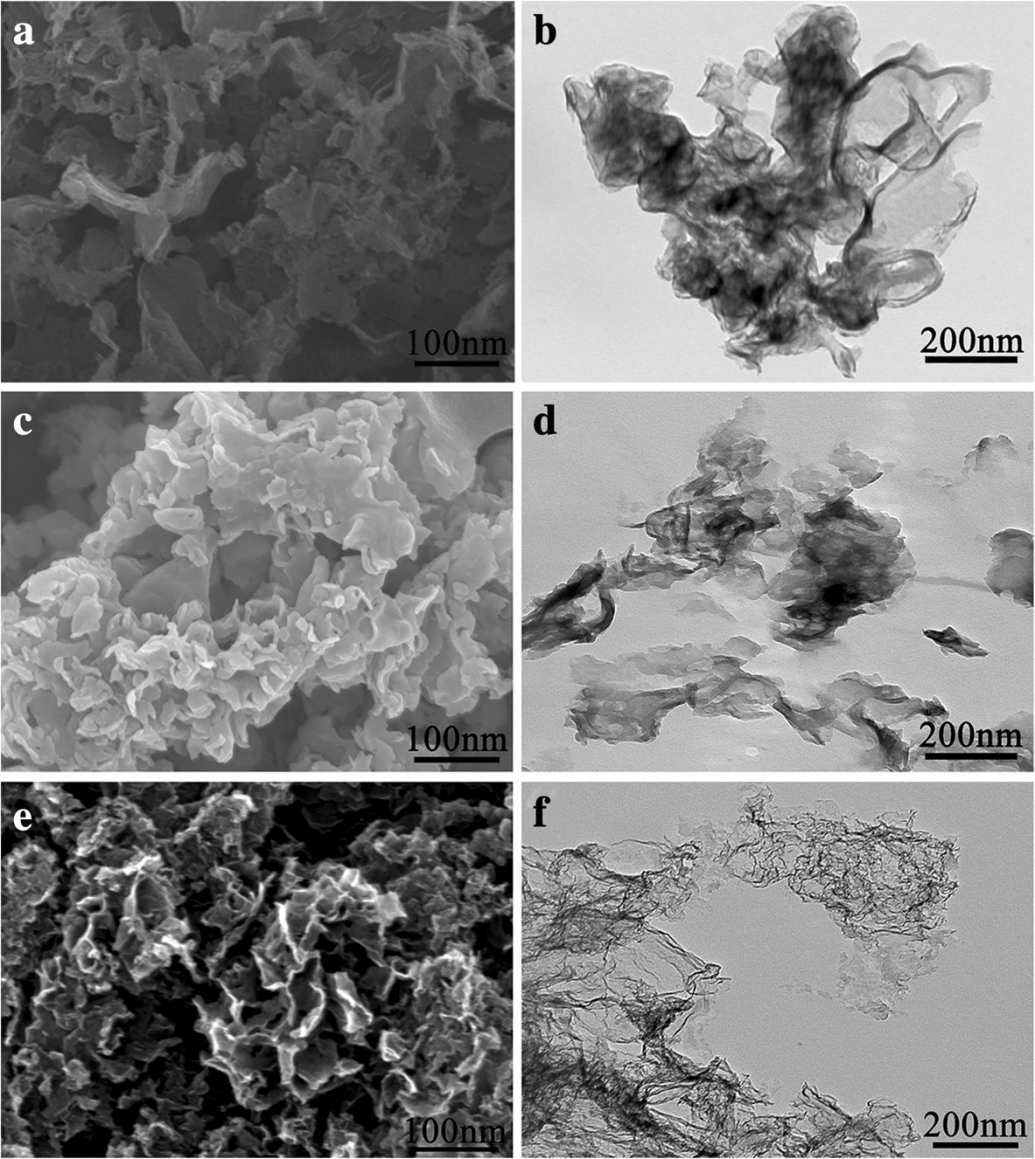
Structural characterizations of samples like carbon nanosheets. **a** SEM and **b** TEM images of g-C_3_N_4_, **c** SEM and **d** TEM images of g-C_3_N_4_/PDA-120, **e** SEM and **f** TEM images of NC-120

In order to determine the morphology of the synthesized samples, SEM and TEM are used for structure observation as shown in Fig. 1. Figure 1a represents the sheet structure of as-synthesized g-C_3_N_4_. The 2D structure of g-C_3_N_4_ is further confirmed from Fig. 1b, which is similar with the previous report [48]. For g-C_3_N_4_/PDA-120 as shown in Fig. 1c, d, the SEM image is similar with that of g-C_3_N_4_. However, the TEM image of g-C_3_N_4_/PDA-120 shows well-dispersed sheet like morphology, compared with as-synthesized g-C_3_N_4_. With the increasing heating temperature from 120 to 160 °C, the thin lamellar structure of carbonized layer could be observed (Additional file 1: Figure S1). After sintering at 900 °C, the SEM images appear honeycomb-like structures as shown in Fig. 1e due to the pyrolysis of g-C_3_N_4_ template, inducing porous carbon structures as shown in Fig. 1f and Additional file 1: Figure S2. The thermo-gravity test of g-C_3_N_4_ was carried out to determine the residue of g-C_3_N_4_, and g-C_3_N_4_ begins to decompose at 520 °C. Under nitrogen protection, fully decomposition is confirmed at 760 °C as shown in Additional file 1: Figure S3. Between 80 and 100 °C, g-C_3_N_4_ will slightly lose its quality due to the evaporation of moisture, and the research result is consistent with previous reports [47]. This indicates that g-C_3_N_4_ could be used as an efficient template for preparing porous carbon structures.

### XRD, FT-IR, and Raman Characterization

**Fig. 2:**
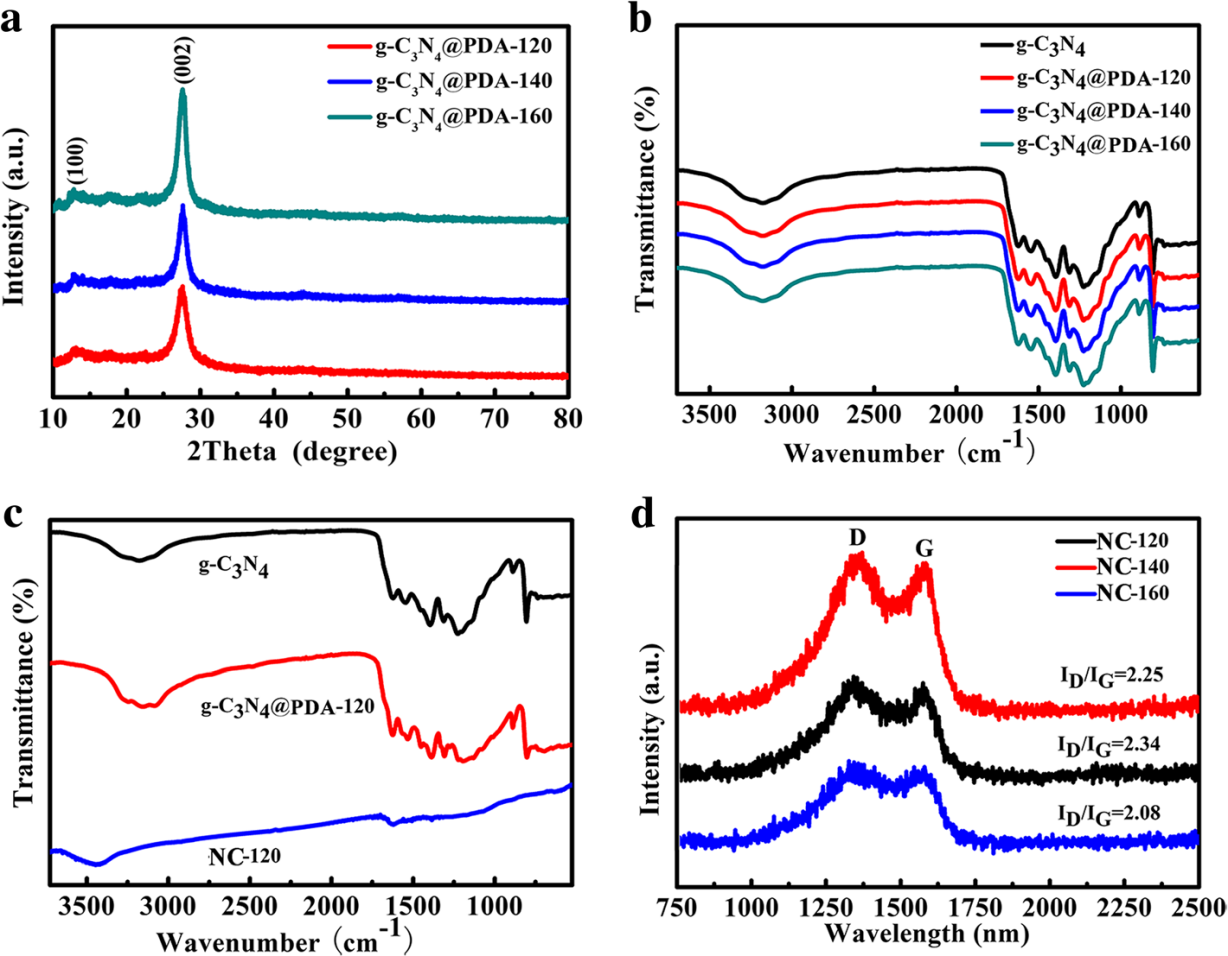
**a** XRD patterns of g-C_3_N_4_@PDA-120, g-C_3_N_4_@PDA-140, and g-C_3_N_4_@PDA-160; **b** FT-IR spectra of g-C_3_N_4_, g-C_3_N_4_@PDA-120, g-C_3_N_4_@PDA-140, and g-C_3_N_4_@PDA-160; **c** FT-IR spectra of g-C_3_N_4_, g-C_3_N_4_/PDA-120, and NC-120; **d** Raman spectra of NC-120, NC-140, and NC-160

Hydrothermal temperature not only affects the structure of samples but also changes the peak of XRD. The three DA-coated g-C_3_N_4_ samples all exhibit two diffraction peaks at 13.0° and 27.4° attributing to (100) and (002) crystal planes of g-C_3_N_4_ template as shown in Fig. 2a. After calcination at 900 °C, the obvious variation for all three samples could be found. The peak at 13.0° vanished, and two new peaks occur around 26.3° and 44.1° relevant to the (002) and (100) planes of graphene, indicating the formation of a new graphitic carbon structure as shown in Additional file 1: Figure S4 [4]. With the increasing hydrothermal temperature, the degree of graphitization and crystalline increases gradually. This is further confirmed by Raman and FT-IR test.

FT-IR spectrometry was performed to analyze the functional groups present on the surfaces of NC-T hybrids. FT-IR spectra of g-C_3_N_4,_ g-C_3_N_4_/PDA-120, g-C_3_N_4_/PDA-140, and g-C_3_N_4_/PDA-160 indicate that these samples include similar carbon and oxygen containing functional groups as shown in Fig. 2b. The peaks between 1200 and 1650 cm^−1^ belong to stretching methods of CN heterocycles, and the broad peaks located in the range of 3000~3500 cm^−1^ are ascribed to the N-H group stretching vibration [49]. With the increasing hydrothermal temperature, the two peaks at 1650 cm^−1^ (C=C) and 3350 cm^−1^ (O-H) are more evident due to DA wrapped on g-C_3_N_4_. After high-temperature carbonization, only three weak peaks remain for NC-120 at 1600 cm^−1^, 1260 cm^−1^, and 3450 cm^−1^ (Fig. 2c), which are ascribed to the C-C and C-N vibration. This indicates that high-temperature carbonization destroys O-H, N-H, and other chemical bonds, while C-N bonds are stable, thus ensuring the effective doping of nitrogen elements.

Raman spectroscopy is employed to evaluate the structural evolution of NC-T. Figure 2d is the Raman spectra of NC-120, NC-140, and NC-160, and the peaks at 1350 and 1580 cm^−1^ correspond to the D and G band, respectively [50, 51]. With the increasing hydrothermal temperature, the intensity ratio of *I*_D_/*I*_G_ decreases from 2.34 to 2.08, indicating the enhanced graphitization degree with the increasing temperature.

### XPS Characterization

**Fig. 3:**
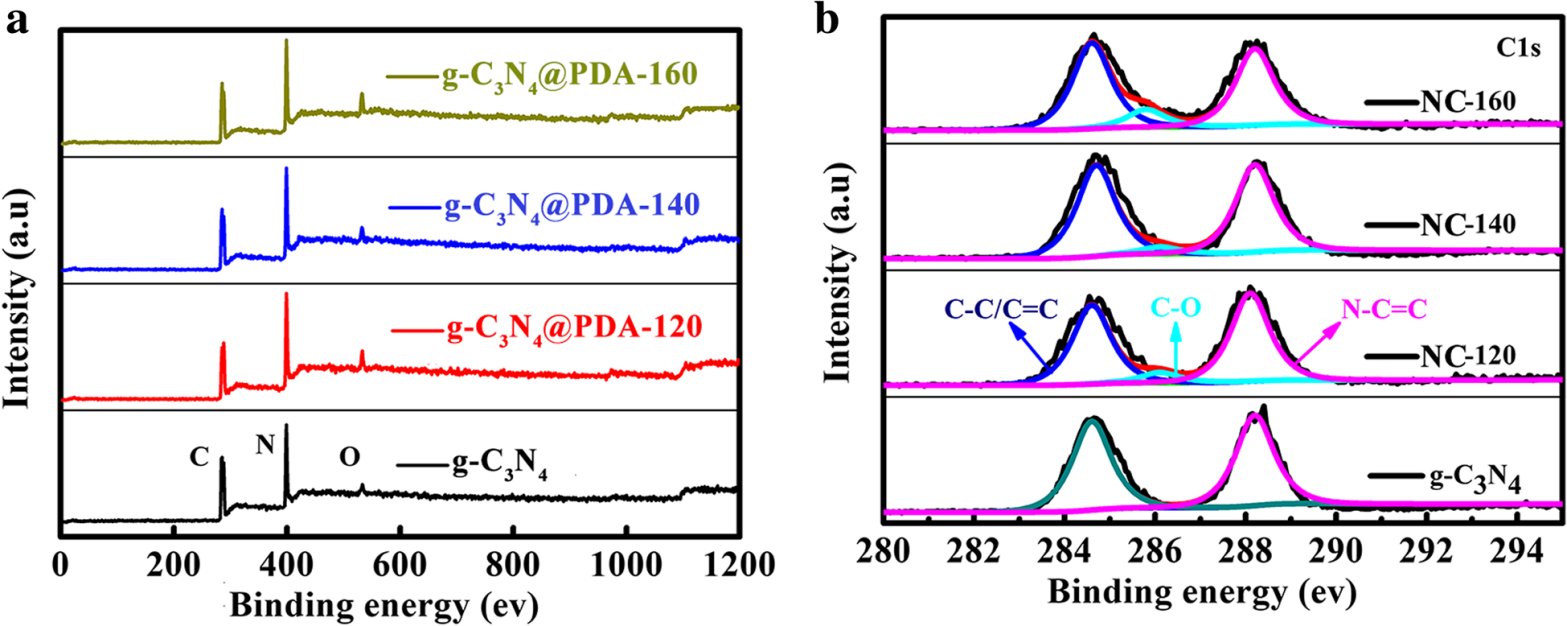
**a** XPS survey and **b** C1s XPS spectra of g-C_3_N_4_@PDA-T prepared at different HT from g-C_3_N_4_ and 120 °C, 140 °C, to 160 °C, respectively

**Fig. 4:**
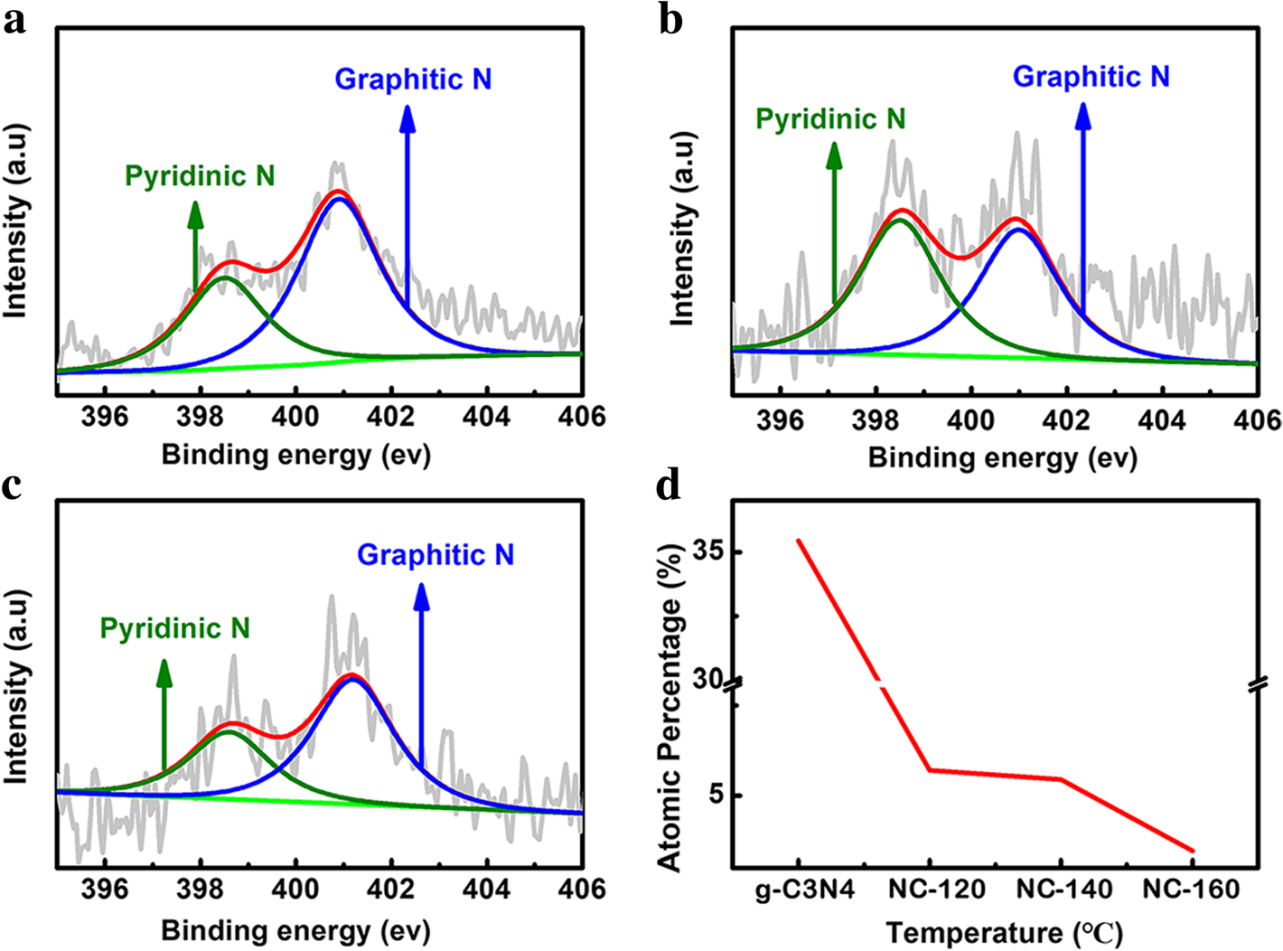
High-resolution XPS spectra of NC-T **a** 120 °C, **b** 140 °C, and **c** 160 °C; **d** The change of absolute atomic content of doped N at different temperature

XPS is used to explore the elemental composition of the samples as shown in Fig. 3. Compared with g-C_3_N_4_, three g-C_3_N_4_/PDA-T samples exhibit increased oxygen content due to the carbonized DA coating on g-C_3_N_4_ (Additional file 1: Table S1). With the increasing hydrothermal temperature from 120 to 160 °C, the N content decreases (Fig. 3a). For C-related peaks, the intensities of C-C/C=C and C-O peaks enhance, while the peak of N-C=C gradually decreases with the increasing hydrothermal temperature as shown in Fig. 3b. According to previous report [24], desirable electrochemical performance could be achieved by samples with high effective N-doping content. Based on XPS spectra of NC-T, C content increases after 900 °C heat treatment with the increase of hydrothermal temperature (Additional file 1: Figure S6). Table 1 gives effective N content of the three samples. The NC-T displays the presence of pyridinic N (398.5 eV) and graphitic N (401.1 eV) [25]. When the hydrothermal temperature is raised to 160 °C, the N content decreased significantly. Figure 4 shows high-resolution N 1 s XPS spectra of NC-T samples. The percentage of pyridinic N and graphitic N as a function of hydrothermal temperature is shown in Fig. 4d. The N content decreases gradually with the increasing hydrothermal temperature.

**Table 1:** The N content in all the samples

Samples	Pyridinic N at %	Graphitic N at %	N content at % (pyridinic N+ graphitic N)
NC-120	2.04	3.67	5.71 at % (2.04 at % + 3.67 at %)
NC-140	2.68	2.65	5.43 at % (2.78 at % + 2.65 at %)
NC-160	1.21	2.24	3.45at % (1.21 at % + 2.24 at %)

### Brunauer–Emmett–Teller (BET) Characterization

The catalytic activity is related to the specific surface area and the pore structure.

The BET specific surface areas of samples are decided by N_2_ adsorption isotherms test at 77 K, and all samples show type IV curves [19]. This implies that the samples have micropores and mesoporous structures as shown in Additional file 1: Figure S5. The specific surface areas of NC-120, NC-140, and NC-160 are 954, 824, and 517 m^2^ g^−1^, respectively, which are significantly higher than those of original g-C_3_N_4_ (85 m^2^ g^−1^). The results show that lower temperature contributes to the formation of large specific surface area and pore size. For the ORR catalyst, the benefit of the layered structure, high specific surface areas, and high nitrogen contents are very significant. The SEM and TEM images of g-C_3_N_4_/PDA-T showed the three samples have a similar-layered structure with pristine g-C_3_N_4_ (Additional file 1: Figure S1). NC-120 exhibits the largest large specific surface area (954 m^2^ g^−1^), and it has a suitable mesoporous structure (≈ 5 nm). The large specific surface area helps to increase the contact area with the reactants and accelerate the reaction [52].

### Electrocatalytic Performance and Discussion

**Fig. 5:**
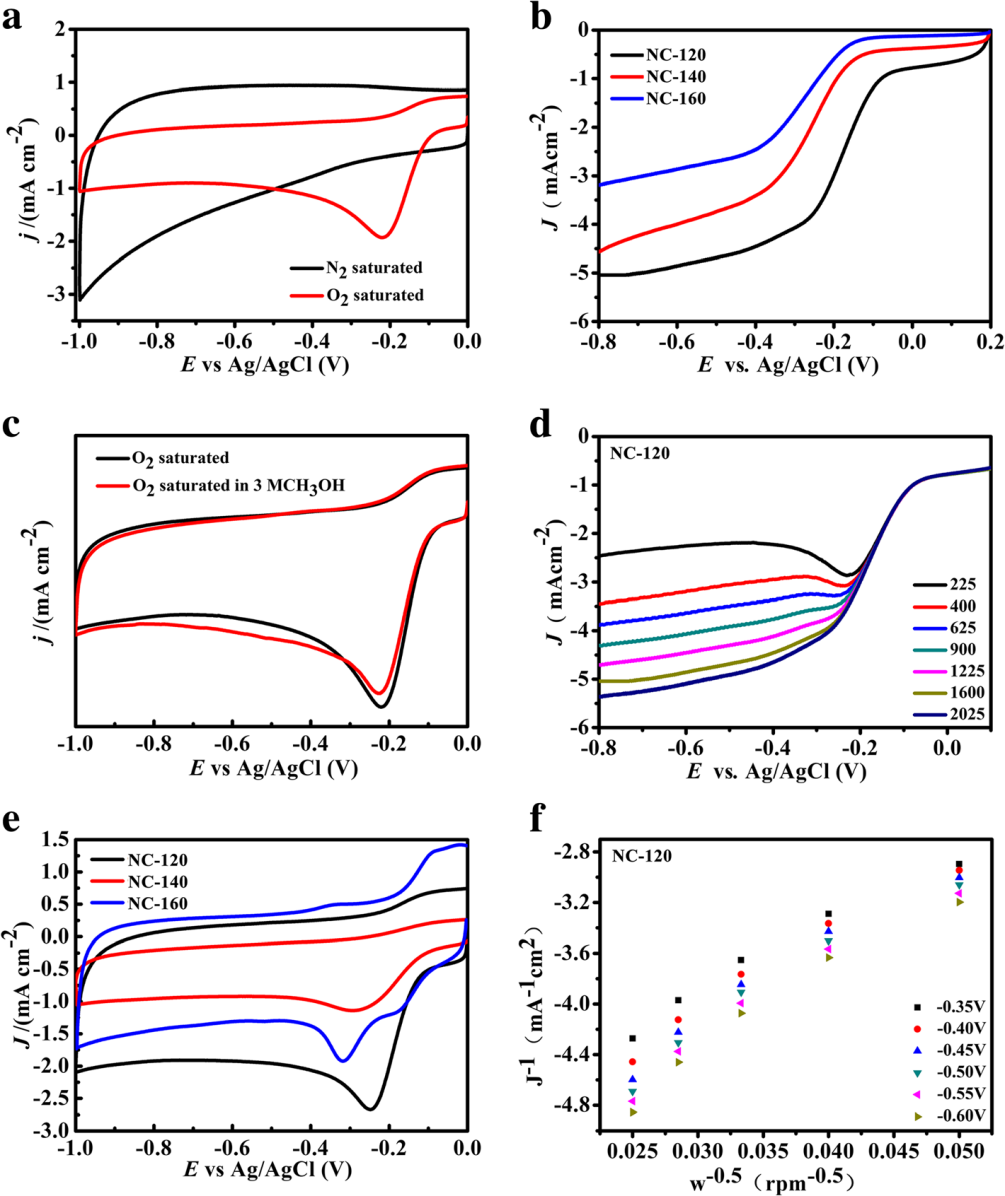
**a** CV curves of NC-120 in N_2_ and O_2_ saturated 0.1 M KOH aqueous solution with a scan rate of 100 mV s^−1^. **b** LSV curves of NC-T at 1600 rmp rotating speeds. **c** Linear polarization curves of NC-120 with different rotation rates at a sweep rate of 5 mV s^−1^ in O_2_-saturated 0.1 M KOH. **d** K-L plots at different potentials based on the results of **c**. **e** CVs of NC-120 in O_2_-saturated 0.1 M KOH solution with 3 M CH_3_OH. **f** CV curves of NC-T in O2 saturated 0.1 M KOH aqueous solution with a scan rate of 100 mV s^−1^

It could be concluded from the above results that NC-120 has the largest specific surface area and the highest nitrogen content, which is very beneficial for ORR [46]. The electrochemical properties of the samples are investigated by cyclic voltammetry (CV) and compared with commercial Pt/C catalysts. The results are shown in Fig. 5 and Table 2. There is no oxygen reduction peak observed for all samples under nitrogen saturated condition (Fig. 5a and Additional file 1: Figure S8). For oxygen saturated condition, there is an obvious oxygen reduction peak, and it becomes more obvious with the decreasing hydrothermal temperature. The results suggest that CV behaviors are associated with the structure of catalysts. In O_2_-saturated 0.1 M KOH solutions, the NC-120 sample expresses the best performance, which is close to Pt-based catalyst and its half-peak potential is 0.224 V, and its ultimate current density is 5.04 mA cm^−1^ (Additional file 1: Figure S7). Figure 5b is the linear scan voltammogram (LSV) curve, showing the limiting current density and onset potentials of the NC-T samples. With the decreasing hydrothermal temperature, the voltage and current density is enhanced, and the performance of catalyst is improved gradually. It can be seen from Fig. 5c that a small movement of that curve is found after the addition of methanol, suggesting that NC-120 has excellent tolerance to methanol. In the Fig. 5(e), the NC-120 exhibited good oxygen reduction properties, which could be due to the large specific surface area and nitrogen content (Additional file 1: Figure S7). For NC-120 (Fig. 5d, f), the number of electron transfer in the process of oxygen reduction is calculated to be 3.9–4.1, indicating that the oxygen reduction reaction of NC-120 catalytic is four electronic processes. NC-120 has the best electrochemical performance, which is attributed to the effective N doping by present strategy.

**Table 2:** Elemental composition, surface area, and electroactivity of samples

Samples	N content at % (pyridinic N+ graphitic N)	S_BET_ (m^2^ g^−1^)	E _half-peak voltage_ (V)	*n*	*J*_L_ (mA cm^−2^)
NC-120	5.71 at % (2.04 at % + 3.67 at %)	954	− 0.224	3.9–4.1	5.040
NC-140	5.43 at % (2.68 at % + 2.65 at %)	833	− 0.257	3.5–3.7	4.072
NC-160	3.45at % (1.21 at % + 2.24 at %)	517	− 0.327	`3.0–3.2	3.165
Pt/C	–	–	− 0.175	4	–

As shown in Scheme 1, DA is used as carbon source and g-C_3_N_4_ nanosheet is used as template and nitrogen source. There are three reasons for choosing this strategy: Firstly, g-C_3_N_4_ can provide multi-layer structure and it could fully disappear at 900 °C. Secondly, DA can provide carbon atom and carbonized DA could cover on both sides of g-C_3_N_4_. PDA was formed on the template of g-C_3_N_4_ in a hydrothermal process using DA as a carbon source. In hydrothermal processes, PDA can strongly adhere to substrate surface of organic or inorganic materials by forming strong covalent and non-covalent bonds on the surface of the substrate. After the coating material is formed, pyrolysis under nitrogen conditions results in the formation of porous carbon materials with large specific surface area. Finally, the decomposed g-C_3_N_4_ would provide N atoms for doping carbon structure, thus effectively increasing active sites for ORR. In previous reports, Liu et al. prepared g-C_3_N_4_@PDA composites by dropping DA solution directly into g-C_3_N_4_ solution under stirring for enhancing visible light photocatalytic H_2_ production activity, which exhibits promising results [37]. In present work, as discussed above, we developed a new strategy to prepare N-doped porous carbon structures with high specific surface area (954 m^2^ g^−1^) and high N content (5.71%) using g-C_3_N_4_ as template and N source simultaneously. The acquired composites exhibit comparable ORR activity, superior durability, and methanol tolerance to Pt/C reference electrocatalyst.

## Conclusion

In summary, we developed a strategy to synthesize N-doped carbon structures. The first step is mainly the hydrothermal process, and the second step is the heat treatment process. With g-C_3_N_4_ as the template and DA as the carbon source, porous carbon networks with high N doping content could be achieved. The resulted network structures can increase the specific surface area of the catalyst and thus provide excellent electrochemical properties including good methanol tolerance effect and stability compared with Pt/C catalyst. The performance of these excellent electrocatalysis may be attributed to the following reasons: (1) N-doped C-layered structure with high content of graphite-N and pyridine-N species provides a highly active site for oxygen reduction reaction. (2) The high specific surface area (954 m^2^ g^−1^) coexists with high graphitic carbon and amorphous carbon, contributing to the electron conduction of the ORR. (3) The porous structures accelerate the transfer of electrons and promote the full utilization of active sites. These advantages all determine the good catalytic effect of this material. In addition, the present method to prepare N-doped carbon-based nanomaterials is economic, eco-friendly, and efficient, making them widely available in fuel cells in future.

## Additional file

Additional file 1: **Figure S1.** (A) SEM and (B) TEM images of g-C_3_N_4_@PDA-120. (C) SEM and (D) TEM images of g-C_3_N_4_@PDA-140. (E) SEM and (F) TEM images of g-C_3_N_4_@PDA-160. **Figure S2.** (A) SEM and (B) TEM images of NC-120. (C) SEM and (D) TEM images of NC-140. (E) SEM and (F) TEM images of NC-160. **Figure S3.** TGA of g-C_3_N_4_ in N_2_ with a temperature rise rate of 5 °C min^−1^. **Figure S4.** XRD patterns of NC-120, NC-140, and NC-160. **Figure S5.** N_2_ adsorption/desorption isotherms (inset, pore size distribution of g-C_3_N_4_ and all NC-T). **Figure S6.** XPS survey of NC-T prepared at different HT from g-C_3_N_4_ and 120, 140, to 160 °C, respectively. **Figure S7.** (a) CV curves of Pt/C and NC-T in N_2_ and O_2_ saturated 0.1 M KOH aqueous solution with a scan rate of 100 mV s^−1^. (b) (c) and (d) Linear polarization curves of NC-T (T = 120°C 140°C 160°C) with different rotation rates at a sweep rate of 5 mV s^−1^in O_2_-saturated 0.1 M KOH. **Figure S8.** CV curves of CN-T in N_2_ and O_2_ saturated 0.1 M KOH aqueous solution with a scan rate of 100 mV s^−1^ (t = 120°C, 140°C, 160°C). Supplementary data related to this article can be found at journal website. (DOC 17806 kb)

## Data Availability

The datasets used or analyzed during the current study are available from the corresponding author on reasonable request.

## Abbreviations

BET: Bunauer–Emmett–Teller
CV: Cyclic voltammetry
DA: Dopamine
g-C_3_N_4_: Graphite carbon nitride
GCE: Glass carbon electrode
LSV: Linear scan voltammogram
ORR: Oxygen reduction reaction
SEM: Scanning electron microscope
TEM: Transmission electron microscope
XPS: X-ray photoelectron spectroscopy

## References

[1] Liao Y, Gao Y, Zhu S, Zheng J, Chen Z, Yin C, Lou X, Zhang D (2015). Facile fabrication of N-doped graphene as efficient electrocatalyst for oxygen reduction reaction. ACS Appl Mater Interfaces.

[2] You C, Jiang X, Wang X, Hua Y, Wang C, Lin Q, Liao S (2018). Nitrogen, sulfur Co-doped carbon derived from naphthalene-based covalent organic framework as an efficient catalyst for oxygen reduction. ACS Appl Mater Interfaces.

[3] Zhang Y, Lu L, Zhang S, Lv Z, Yang D, Liu J, Chen Y, Tian X, Jin H, Song W (2018). Biomass chitosan derived cobalt/nitrogen doped carbon nanotubes for the electrocatalytic oxygen reduction reaction. J Mater Chem A.

[4] Li Q, Xu D, Ou X, Yan F (2017). Nitrogen-doped graphitic porous carbon nanosheets derived from in situ formed g-c3 n4 templates for the oxygen reduction reaction. Chem Asian J.

[5] Kong A, Dong B, Zhu X, Kong Y, Zhang J, Shan Y (2013). Ordered mesoporous Fe-porphyrin-like architectures as excellent cathode materials for the oxygen reduction reaction in both alkaline and acidic media. Chem.

[6] Cui X, Yang S, Yan X, Leng J, Shuang S, Ajayan PM, Zhang Z (2016). Pyridinic-nitrogen-dominated graphene aerogels with Fe-N-C coordination for highly efficient oxygen reduction reaction. Adv Funct Mater.

[7] Chen Z, Lu S, Wu Q, He F, Zhao N, He C, Shi C (2018). Salt-assisted synthesis of 3D open porous g-C3N4 decorated with cyano groups for photocatalytic hydrogen evolution. Nanoscale.

[8] Chen P, Wang L-K, Wang G, Gao M-R, Ge J, Yuan W-J, Shen Y-H, Xie A-J, Yu S-H (2014). Nitrogen-doped nanoporous carbon nanosheets derived from plant biomass: an efficient catalyst for oxygen reduction reaction. Energy Environ. Sci..

[9] Long G, Li X, Wan K, Liang Z, Piao J, Tsiakaras P (2017). Pt/C N-doped electrocatalysts: Superior electrocatalytic activity for methanol oxidation reaction and mechanistic insight into interfacial enhancement. Appl Catal B-Environ.

[10] Liu J, Huang Y (2018). Oxygen reduction reaction on PtCo nanocatalyst: (Bi)sulfate anion poisoning. Nanoscale Res Lett.

[11] Zhou X, Qiao J, Yang L, Zhang J (2014). A review of graphene-based nanostructural materials for both catalyst supports and metal-free catalysts in PEM fuel cell oxygen reduction reactions. Adv Energy Mater.

[12] Chen Z, Higgins D, Yu A, Zhang L, Zhang J (2011). A review on non-precious metal electrocatalysts for PEM fuel cells. Energy Environ Sci.

[13] Zeng H, Wang W, Li J, Luo J and Chen S. (2018) In situ generated dual-template method for Fe/N/S co-doped hierarchically porous honeycomb carbon for high-performance oxygen reduction. ACS Appl Mater Interfaces 10:8721-8729. 10.1021/acsami.7b19645 29481037

[14] Xuan C-J, Hou B-S, Xia W-W, Peng Z-K, Shen T, Xin HL, Zhang G, Wang D (2018). From zif-8 polyhedron to three-dimensional nitrogen doped hierarchical porous carbon: an efficient electrocatalyst for oxygen reduction reaction. J Mater Chem A.

[15] Sun T, Xu L, Li S, Chai W, Huang Y, Yan Y, Chen J (2016). Cobalt-nitrogen-doped ordered macro-/mesoporous carbon for highly efficient oxygen reduction reaction. Appl Catal B-Environ.

[16] Ong WJ, Tan LL, Ng YH, Yong ST, Chai SP (2016). Graphitic carbon nitride (g-c3n4)-based photocatalysts for artificial photosynthesis and environmental remediation: are we a step closer to achieving sustainability?. Chem Rev.

[17] Dai L, Xue Y, Qu L, Choi HJ, Baek JB (2015). Metal-free catalysts for oxygen reduction reaction. Chem Rev.

[18] Wang C, Zhang K, Xu H, Du Y, Goh MCJJ (2019). Anchoring gold nanoparticles on poly(3,4-ethylenedioxythiophene) (PEDOT) nanonet as three-dimensional electrocatalysts toward ethanol and 2-propanol oxidation. J Colloid Interface Sci.

[19] Zhang J, Zhao Z, Xia Z, Dai LJNN (2015). A metal-free bifunctional electrocatalyst for oxygen reduction and oxygen evolution reactions. Nature Nanotech.

[20] Zhang Z, Sun J, Dou M, Ji J, Wang F, JAAM and Interfaces (2017). Nitrogen and phosphorus codoped mesoporous carbon derived from polypyrrole as superior metal-free electrocatalyst towards the oxygen reduction reaction. ACS Appl Mater Interfaces.

[21] Jin L, Xu H, Chen C, Song T, Wang C, Wang Y, Shang H, Du Y (2019). Uniform PdCu coated Te nanowires as efficient catalysts for electrooxidation of ethylene glycol. J Colloid Interface Sci.

[22] Wang Q, Hu W-H, Huang Y-H (2017). Nitrogen doped graphene anchored cobalt oxides efficiently bi-functionally catalyze both oxygen reduction reaction and oxygen revolution reaction. Int J Hydrogen Energy.

[23] Lv Q, Si W, Yang Z, Wang N, Tu Z, Yi Y, Huang C, Jiang L, Zhang M, He J, Long Y (2017). Nitrogen-doped porous graphdiyne: a highly efficient metal-free electrocatalyst for oxygen reduction reaction. ACS Appl Mater Interfaces.

[24] Huang J, Han J, Gao T, Zhang X, Li J, Li Z, Xu P, Song B (2017). Metal-free nitrogen-doped carbon nanoribbons as highly efficient electrocatalysts for oxygen reduction reaction. Carbon.

[25] Zhang XY, Sun SH, Sun XJ, Zhao YR, Chen L, Yang Y, Lu W, Li DB (2016). Plasma-induced, nitrogen-doped graphene-based aerogels for high-performance supercapacitors. Light Sci Appl.

[26] Fan M, Zhu C, Yang J, Sun D (2016). Facile self-assembly N-doped graphene quantum dots/graphene for oxygen reduction reaction. Electrochim Acta.

[27] Lee S, Lee Y-W, Kwak D-H, Lee J-Y, Han S-B, Sohn JI, Park K-W (2016). Three-dimensional porous metal–nitrogen doped carbon nanostructure as a superior non-precious electrocatalyst in oxygen reduction reaction. J Ind Eng Chem.

[28] Jia Y, Sun X, Shi Z, Jiang K, Liu H, Ben J, Li D (2018). Modulating the surface state of SiC to control carrier transport in graphene/SiC. Small.

[29] Li Q, Chen W, Xiao H, Gong Y, Li Z, Zheng L, Zheng X, Yan W, Cheong WC, Shen R, Fu N, Gu L, Zhuang Z, Chen C, Wang D, Peng Q, Li J, Li Y (2018). Fe isolated single atoms on S, N codoped carbon by copolymer pyrolysis strategy for highly efficient oxygen reduction reaction. Adv Mater.

[30] Kone I, Xie A, Tang Y, Chen Y, Liu J, Chen Y, Sun Y, Jin YX, Wan PJAAMI (2017). Hierarchical porous carbon doped with iron-nitrogen-sulfur for efficient oxygen reduction reaction. ACS Appl. Mater. Interfaces.

[31] Guo D, Shibuya R, Akiba C, Saji S, Kondo T (2016). Active sites of nitrogen-doped carbon materials for oxygen reduction reaction clarified using model catalysts. Sci.

[32] Yan X, Yao Y, Chen Y (2018). Highly active and stable Fe-N-C oxygen reduction electrocatalysts derived from electrospinning and in situ pyrolysis. Nanoscale Res Lett.

[33] Zan Y, Zhang Z, Liu H, Dou M, Wang F (2017). Nitrogen and phosphorus co-doped hierarchically porous carbons derived from cattle bones as efficient metal-free electrocatalysts for the oxygen reduction reaction. J Mater Chem A.

[34] Ma R, Zhou Y, Li P, Chen Y, Wang J, Liu Q (2016). Self-assembly of nitrogen-doped graphene-wrapped carbon nanoparticles as an efficient electrocatalyst for oxygen reduction reaction. Electrochim Acta.

[35] Li J, Zhang Y, Zhang X, Huang J, Han J, Zhang Z, Han X, Xu P, Song B (2017). S, N dual-doped graphene-like carbon nanosheets as efficient oxygen reduction reaction electrocatalysts. ACS Appl Mater Interfaces.

[36] Li J, Zhang Y, Zhang X, Han J, Wang Y, Gu L, Zhang Z, Wang X, Jian J, Xu P, Song B (2015). Direct transformation from graphitic C3N4 to nitrogen-doped graphene: an efficient metal-free electrocatalyst for oxygen reduction reaction. ACS Appl Mater Interfaces.

[37] Liu M, Cheng B, Yu J, Zhang L (2018). Dopamine modified g-C3N4 and its enhanced visible-light photocatalytic H_2_-production activity ACS Sustain. Chem Eng.

[38] Wu Y, Wang J, Muhammad Y, Subhan S, Zhang Y, Ling Y, Li J, Zhao Z-X, Zhao Z-X (2018). Pyrrolic N-enriched carbon fabricated from dopamine-melamine via fast mechanochemical copolymerization for highly selective separation of CO_2_ from CO_2_/N_2_. Chem Eng J.

[39] Thurston JH, Hunter NM, Cornell KA (2016). Preparation and characterization of photoactive antimicrobial graphitic carbon nitride (g-C3N4) films. RSC Adv.

[40] Yin Y, Wu P, Zhang H, Cai C (2014). Nitrogen/carbon atomic ratio-dependent performances of nitrogen-doped carbon-coated metal oxide nanocrystals for anodes in lithium-ion batteries. ACS Appl Mater Interfaces.

[41] Li Y, Fang L, Jin R, Yang Y, Fang X, Xing Y, Song S (2015). Preparation and enhanced visible light photocatalytic activity of novel g-C3N4 nanosheets loaded with Ag2CO3 nanoparticles. Nanoscale.

[42] Li DB, Sun XJ, Jia YP, Stockman MI, Paudel HP, Song H, Jiang H, Li ZM (2017). Direct observation of localized surface plasmon field enhancement by Kelvin probe force microscopy. Light Sci Appl.

[43] Ferrero GA, Fuertes AB, Sevilla M (2015). N-doped porous carbon capsules with tunable porosity for high-performance supercapacitors. J Mater Chem A.

[44] Yadav RM, Wu J, Kochandra R, Ma L, Tiwary CS, Ge L, Ye G, Vajtai R, Lou J, Ajayan PM (2015). Carbon nitrogen nanotubes as efficient bifunctional electrocatalysts for oxygen reduction and evolution reactions. ACS Appl Mater Interfaces.

[45] Bayram E, Yilmaz G, Mukerjee S (2016). A solution-based procedure for synthesis of nitrogen doped graphene as an efficient electrocatalyst for oxygen reduction reactions in acidic and alkaline electrolytes. Appl Catal B-Environ.

[46] Gao F, Zhang Y, Song P, Wang J, Wang C, Guo J, Du Y (2019). Self-template construction of Sub-24 nm PdeAg hollow nanodendrites as highly efficient electrocatalysts for ethylene glycol oxidation. J Power Sources.

[47] Yu H, Shang L, Bian T, Shi R, Waterhouse GIN, Zhao Y, Zhou C, Wu L-Z, Tung C-H, Zhang T (2016). Nitrogen-doped porous carbon nanosheets templated from g-c3n4as metal-free electrocatalysts for efficient oxygen reduction reaction. Adv Mater.

[48] Dong G, Zhao K, Zhang L (2012). Carbon self-doping induced high electronic conductivity and photoreactivity of g-C3N4. Chem Commun (Camb).

[49] Yan SC, Li ZS, Zou ZG (2009). Photodegradation performance of g-C3N4 fabricated by directly heating melamine. Langmuir.

[50] Chen H, Sun F, Wang J, Li W, Qiao W, Ling L, Long D (2013). Nitrogen doping effects on the physical and chemical properties of mesoporous carbons. J Physl Chem C.

[51] Kudin KN, Ozbas B, Schniepp HC, Prud’homme RK, Aksay IA, Car R (2008). Raman spectra of graphite oxide and functionalized graphene sheets. Nano lett.

[52] Zhou G, Kim N-R, Chun S-E, Lee W, Um M-K, Chou T-W, Islam MF, Byun J-H, Oh Y (2018). Highly porous and easy shapeable poly-dopamine derived graphene-coated single walled carbon nanotube aerogels for stretchable wire-type supercapacitors. Carbon.

