# Supplementary material for: Porous Carbon Networks Derived From Graphitic Carbon Nitride for Efficient Oxygen Reduction Reaction
Source: Nanoscale Res Lett. 2019 Jul 24;14:249. doi: 10.1186/s11671-019-3073-0 (PMC6656852; doi:10.1186/s11671-019-3073-0)
Supplement: Supplementary file 1 — Figure S1. (A) SEM and (B) TEM images of g-C3N4@PDA-120. (C) SEM and (D) TEM images of g-C3N4@PDA-140. (E) SEM and (F) TEM images of g-C3N4@PDA-160. Figure S2. (A) SEM and (B) TEM images of NC-120. (C) SEM and (D) TEM images of NC-140. (E) SEM and (F) TEM images of NC-160. Figure S3. TGA of g-C3N4 in N2 with a temperature rise rate of 5 °C min−1. Figure S4. XRD patterns of NC-120, NC-140, and NC-160. Figure S5. N2 adsorption/desorption isotherms (inset, pore size distribution of g-C3N4 and all NC-T). Figure S6. XPS survey of NC-T prepared at different HT from g-C3N4 and 120, 140, to 160 °C, respectively. Figure S7. (a) CV curves of Pt/C and NC-T in N2 and O2 saturated 0.1 M KOH aqueous solution with a scan rate of 100 mV s−1. (b) (c) and (d) Linear polarization curves of NC-T (T = 120°C 140°C 160°C) with different rotation rates at a sweep rate of 5 mV s−1in O2-saturated 0.1 M KOH. Figure S8. CV curves of CN-T in N2 and O2 saturated 0.1 M KOH aqueous solution with a scan rate of 100 mV s−1 (t = 120°C, 140°C, 160°C). Supplementary data related to this article can be found at journal website. (DOC 17806 kb) [file 11671_2019_3073_MOESM1_ESM.doc]

Supplementary Material

**Nitrogen-Doped Carbon Networks derived from g-C_3_N_4_ Template for Efficient Oxygen Reduction Reaction**

*Chenxia Li^a^, Xuesong Li^a*^, Xiaojuan Sun^b^, Xueyu Zhang^a^, Lianfeng Duan^a^, Xijia Yang^a^, Liying Wang^a^, Wei Lü^a*^*

^a^Key Laboratory of Advanced Structural Materials, Ministry of Education & Advanced Institute of Materials Science, Changchun University of Technology, Changchun, Changchun 130012, China

^b^State Key Laboratory of Luminescence and Applications, Changchun Institute of

Optics, Fine Mechanics and Physics, Chinese Academy of Sciences, Changchun

130012, China

Characterization: The structure of the NC-T was analyzed by X-ray diffraction (XRD) (D-MAX II A X-ray diffractometer). Transmission electron microscopy (TEM) (Tecnai F20) and Scanning electron microscope (SEM) (JEOL7610) was used to observe the morphology and structure. Raman (Horiba, Japan) and Fourier transform infrared (FT-IR) (Nicolet iS50) spectra can obtain information about the structure and properties of molecules. The chemical composition and characteristics of products were measured by X-ray photoelectron spectroscopy (XPS) (Kratos Axis UltraDLD, Japan). Specific surface areas and pore size distributions were determined from N_2_ adsorption-desorption (77K) isotherms on a Micromeritics ASAP 2020 instrument (MICROSENSOR, USA). Cyclic voltammetry (CV) (HUA CHEN 660`e) analysis was measured in the voltage range of -1 to 0 V with scan rate of 100 mVs^-1^. Rotating disk electrode (RDE) measurements were conductedwith different rotation speed from 225 to 2025 rpm at a scan rate of 5mVs ^-1^ . The electron transfer number can be obtained based on theKoutecky-Levich (K-L) equations:


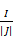
 =
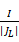
 +
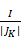
 =
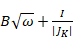
 = 0.2n*FC_0_(D_0_*)^2/3^
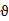
-1/6

J: The measured current;

J_L_: The diffusion limiting current;

J_K_: The kinetic current density;

ω:The rotation speed in rpm;

F: The Faraday constant (96485C mol^-1^ );

C_0_: The bulk concentration of oxygen (1.2x10^-6^ mol cm^-1^ );

D_0_: The diffusion coefficient of oxygen in 0.1 M KOH (1.9x10^-5^cm^2^s^-1^ );


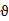
: The kinetic viscosity (0.01 cm^2^s^-1^ );

n: The electron transfer number in ORR.(n can be calculated from the slope of the K-L plots).


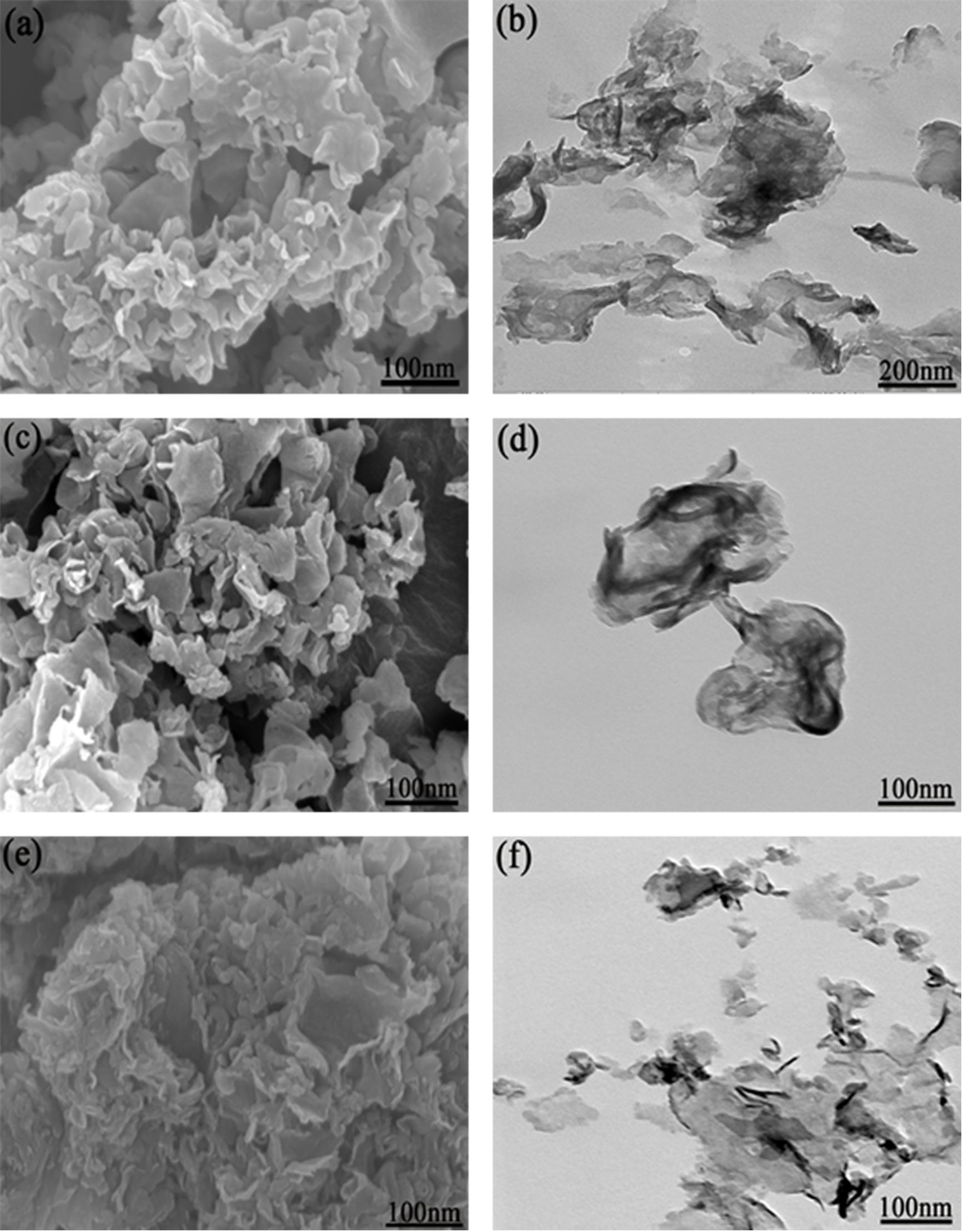


Figure S1 (A) SEM and (B) TEM images of g-C_3_N_4_@PDA-120. (C) SEM and (D) TEM images of g-C_3_N_4_@PDA-140. (E) SEM and (F) TEM images of g-C_3_N_4_@PDA-160.


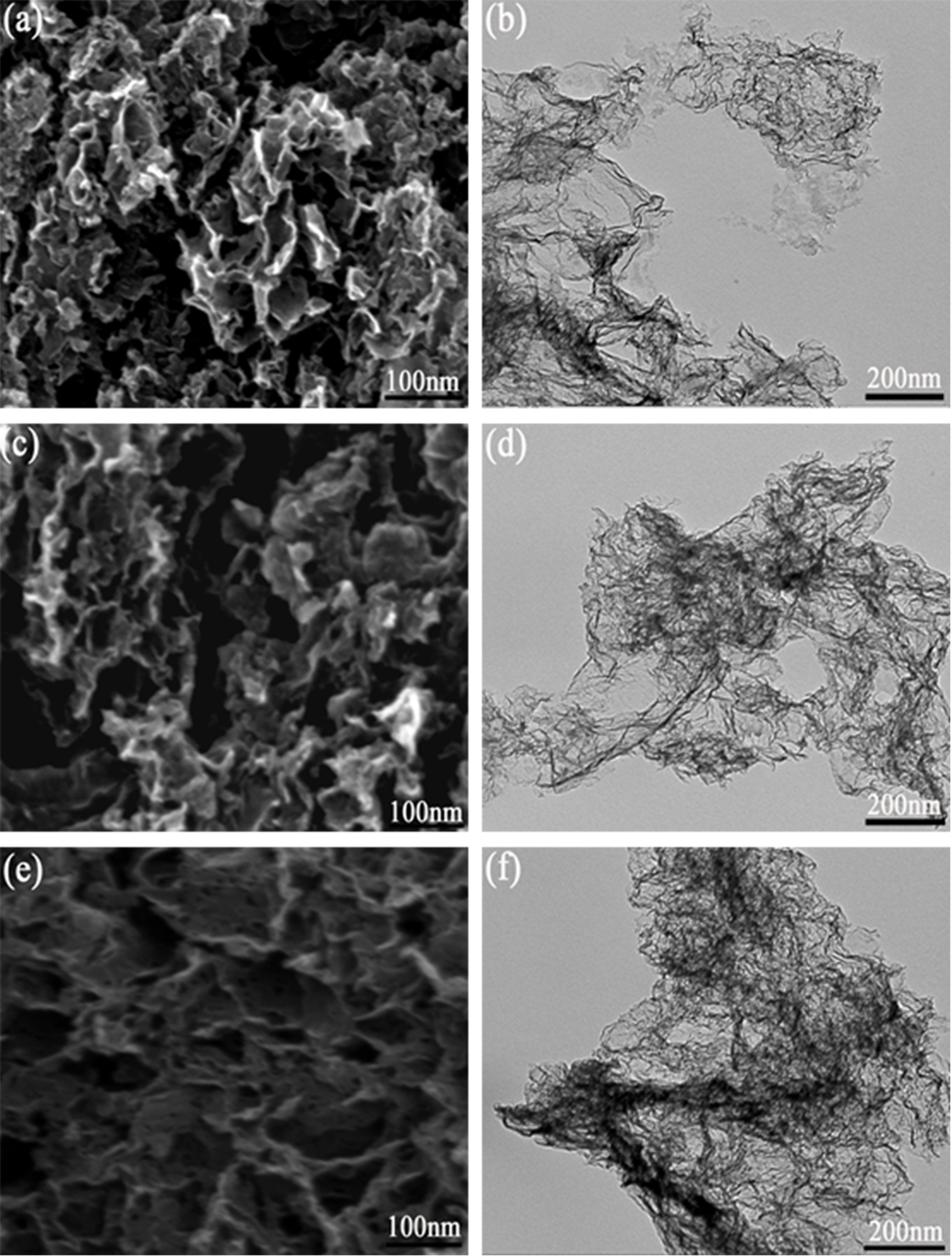


Figure S2 (A) SEM and (B) TEM images of NC-120. (C) SEM and (D) TEM images of NC-140. (E) SEM and (F) TEM images of NC-160.


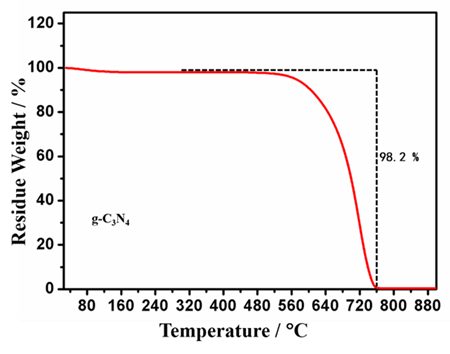


Figure S3 TGA of g-C_3_N_4_ in N_2_ with a temperature rise rate of 5 °C min^-1^.


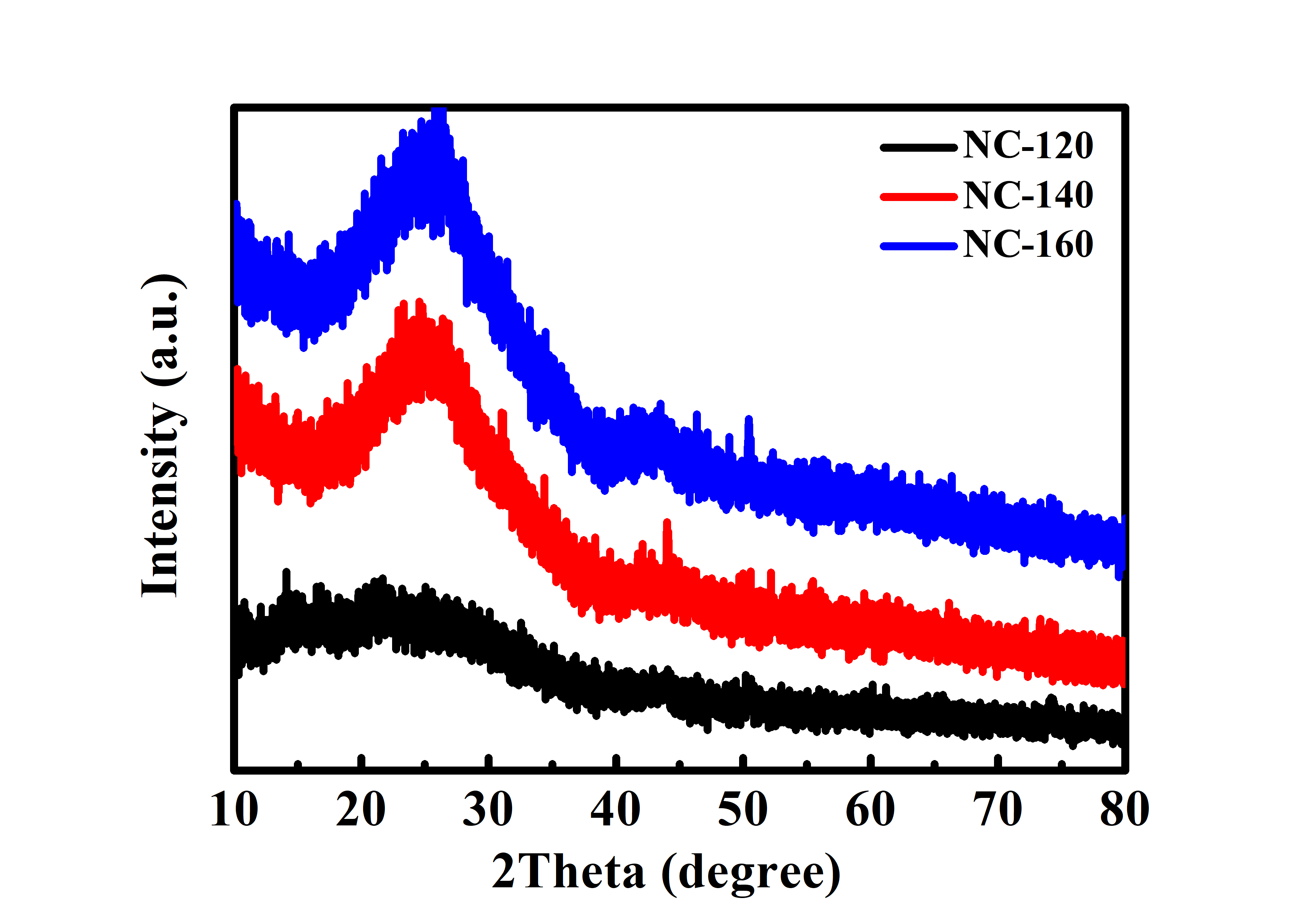


Figure S4 XRD patterns of NC-120, NC-140, NC-160.


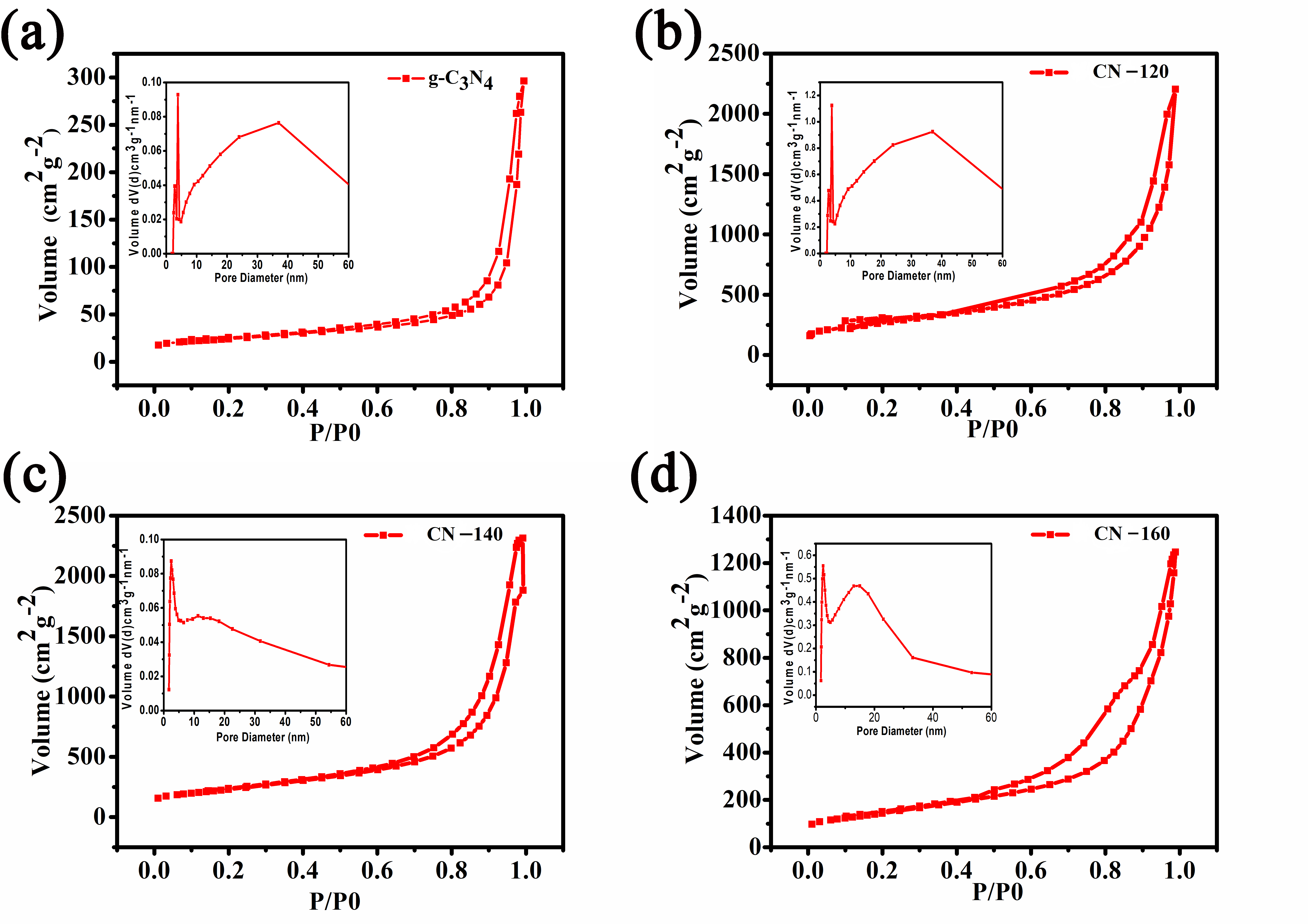


**Figure S5** N_2_ adsorption/desorption isotherms (inset, pore size distribution of g-C_3_N_4_ and all NC-T.)


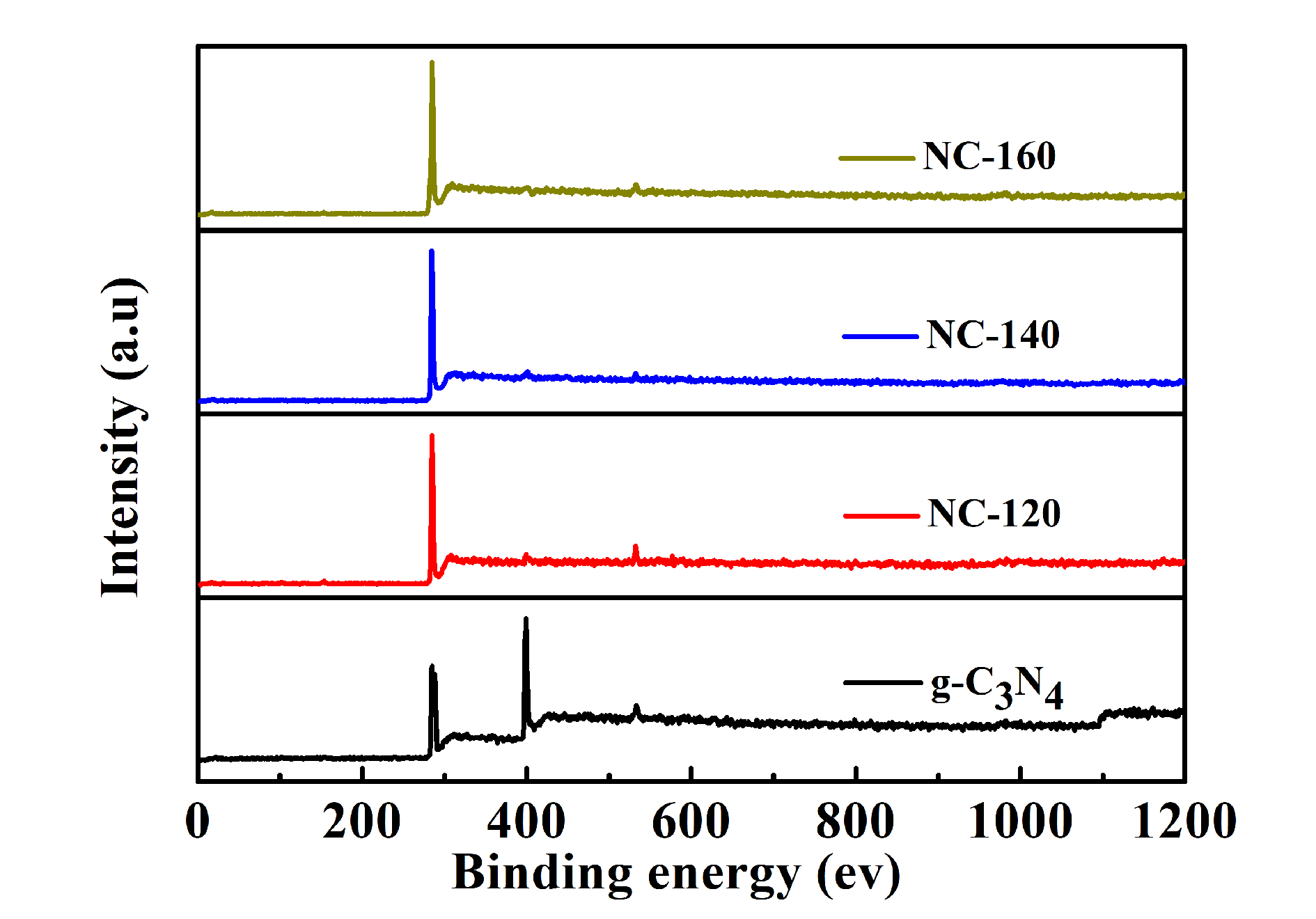


Figure S6 XPS survey of NC-T prepared at different HT from g-C_3_N_4_ and 120, 140 to 160 °C, respectively

Table S1 The element compositions in all the sample

| samples | C content  at % | N content  at % | O content  at % |
| --- | --- | --- | --- |
| g-C_3_N_4_ | 59.82 at % | 35.43 at % | 4.76 at % |
| g-C_3_N_4_@PDA-120 | 57.57 at % | 36.17 at % | 6.26 at % |
| g-C_3_N_4_@PDA-140 | 57.77 at % | 35.8 at % | 7.55 at % |
| g-C_3_N_4_@PDA-160 | 57.95 at % | 33.68 at % | 8.37 at % |


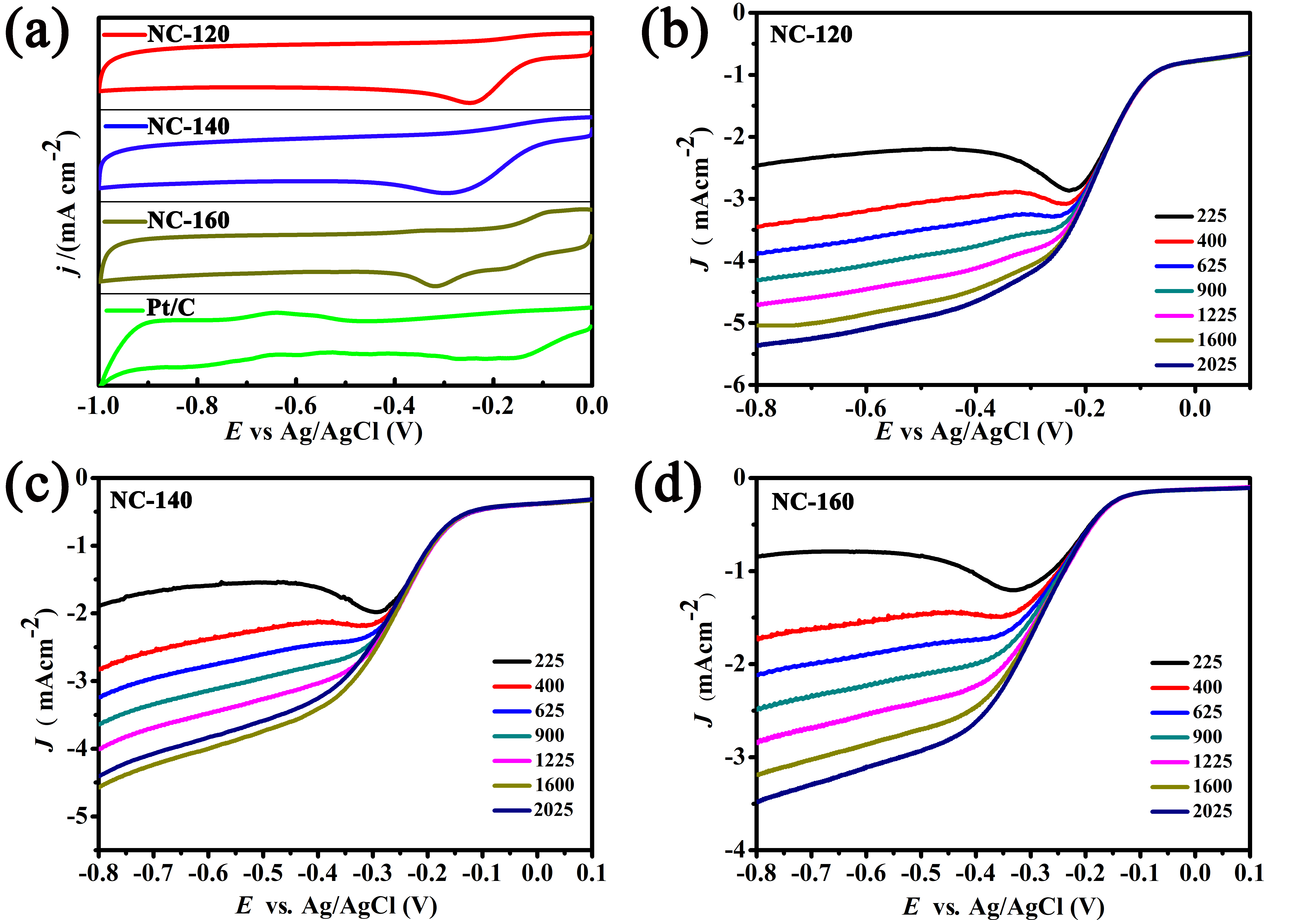


Figure S7 (a) CV curves of Pt/C and NC-T in N_2_ and O_2_ saturated 0.1 M KOH aqueous solution with a scan rate of 100 mV s^−1^. (b) (c) and (d) Linear polarization curves of NC-T (T=120℃ 140℃ 160℃) with different rotation rates at a sweep rate of 5 mV s^−1^in O_2_-saturated 0.1 M KOH


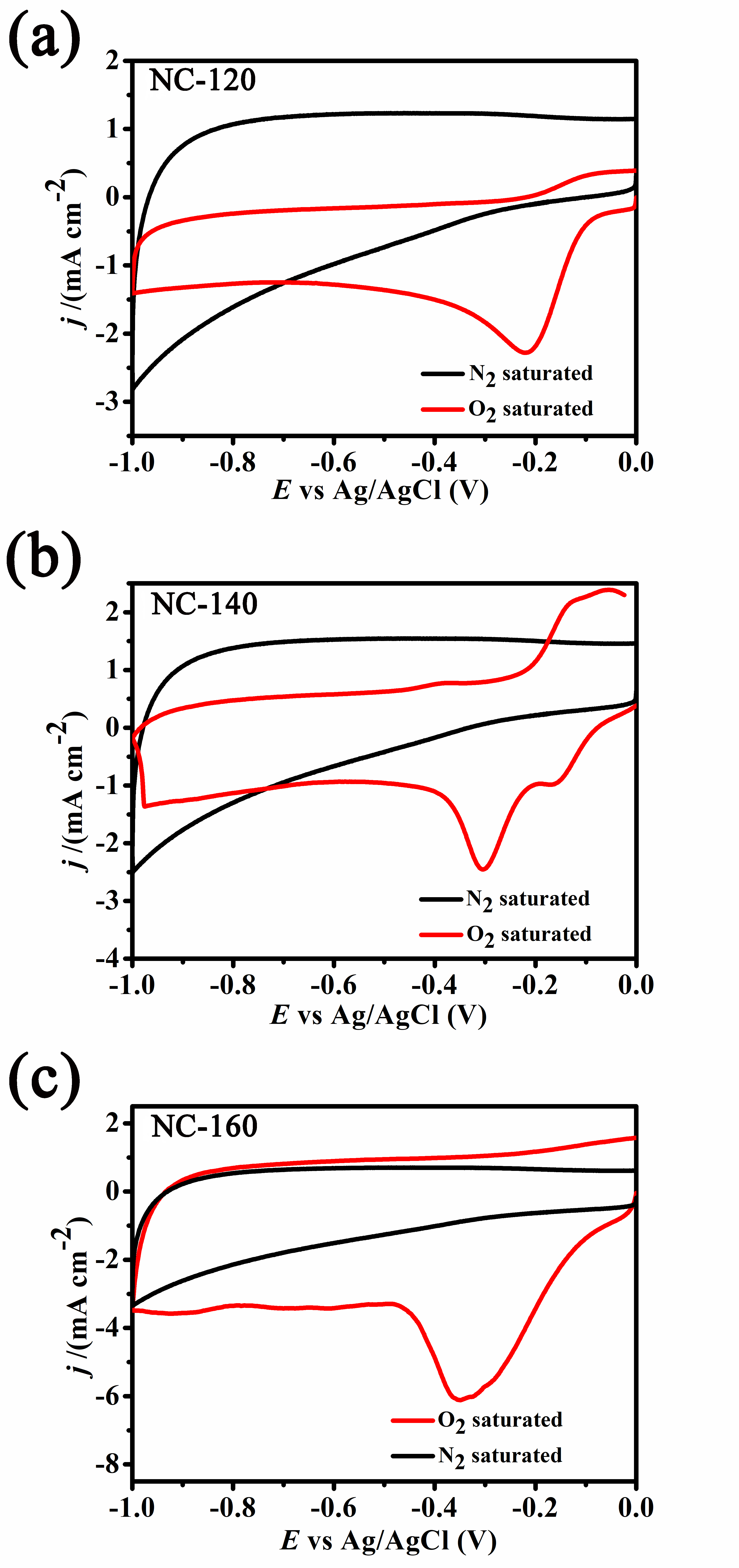


Figure S8 CV curves of CN-T in N_2_ and O_2_ saturated 0.1 M KOH aqueous solution with a scan rate of 100 mV s^−1^ (t=120℃, 140℃, 160℃)
